# Yunpi Qufeng Chushi Formula for Pre-Rheumatoid Arthritis: Study Protocol for a Multiple-Center, Double-Blind, Placebo-Controlled Randomized Controlled Trial

**DOI:** 10.3389/fphar.2022.793394

**Published:** 2022-02-14

**Authors:** Yujun Tang, Haichang Li, Lin Huang, Qiao Wang, Yongmei Han, Huaxiang Wu, Xiao Su, Xiujuan Hou, Chuanbing Huang, Changsong Lin, Qingwen Tao, Jinyang Tang, Wei Cao, Zhijun Xie, Chengping Wen

**Affiliations:** ^1^ College of Basic Medical Science, Zhejiang Chinese Medical University, Hangzhou, China; ^2^ Department of Rheumatology, Sir Run Run Shaw Hospital, Zhejiang University, School of Medicine, Hangzhou, China; ^3^ The Second Affillated Hospital Zhejiang University School of Medicine, Hangzhou, China; ^4^ Shanghai Municipal Hospital of Traditional Chinese Medicine, Shanghai University of Traditional Chinese Medicine, Shanghai, China; ^5^ Division of Rheumatology, Dong Fang Hospital, Beijing University of Chinese Medicine, Beijing, China; ^6^ Department of Rheumatology, The First Affiliated Hospital of Anhui University of Chinese Medicine, Hefei, China; ^7^ Department of Rheumatology, The First Affiliated Hospital of Guangzhou University of Chinese Medicine, Guangzhou, China; ^8^ China-Japan Friendship Hospital, Beijing, China; ^9^ Xiyuan Hospital of China Academy of Chinese Medicine Science, Beijing, China; ^10^ Wangjing Hospital of China Academy of Chinese Medical Sciences, Beijing, China

**Keywords:** pre-rheumatoid arthritis, randomized controlled trial, double-blind, Chinese medicine, placebo, protocol

## Abstract

**Introduction:** Rheumatoid arthritis (RA) is an autoimmune disease characterized by progressive bone erosion on diarthrodial joints. RA patients usually experienced three stages before final diagnosis: the health period, the pre-clinical period (immune response exists without clinical symptoms), and the pre-RA period (immune response exists with mild inflammatory manifestation). Presently, there is seldom guidance referring to early intervention which is a benefit for stable disease conditions and low morbidity. Prophylactic treatment is a major feature of traditional Chinese medicine (TCM). In this present study, a multi-center, double-blind, placebo-controlled clinical trial is carried out to evaluate both efficacy and safety in preventing RA progression on Yunpi Qufeng Chushi formula (YQCF).

**Method:** The multi-center, double-blind, placebo-controlled clinical trial is conducted in 13 hospitals nationwide. A total of 390 patients ages between 18 and 70 will be recruited in the trial. They will be randomly assigned to the intervention group (YQCF) and placebo group. The follow-up visit will be taken every 3 months from baseline to 1 year. Diagnosis, disease activity scores, clinical disease activity index (CDAI), simplified disease activity index (SDAI), TCM syndrome scores, and safety assessments will be recorded at every visit. Joint color doppler ultrasound, health assessment questionnaire-disability index (HAQ-DI), and functional assessment of chronic illness therapy-fatigue (FACIT-F) will be recorded at baseline and the last visit.

**Discussion:** This work will provide evidence of YQCF in preventing RA progression. However, whether early intervention would benefit the controlling RA disease still needs a long-term follow-up.

**Ethics and dissemination:** Protocol version 2 (201910-1). This research was approved by the medical ethics committee of Zhejiang Chinese Medical University (2019-045). Results will be published in a peer-reviewed academic journal.

**Trial registration numbers:** http://www.chictr.org.cn/index.aspx, ChiCTR1900024166.

## 1 Introduction

Rheumatoid arthritis (RA) is an autoimmune disease characterized by progressive bone erosion on diarthrodial joints. The global incidence of RA is about 0.5–1% (Firestein et al., 2020). Although the pathogenesis of RA is not fully understood, genes, infection, and the environment have been demonstrated to be involved. RA patients usually experienced three stages before final diagnosis, namely the health period, the pre-clinical period (immune response exists without clinical symptoms), and the pre-RA period (immune response exists with mild inflammatory manifestation), and sometimes making a definite diagnosis is time-consuming (Smolen et al., 2018).

RA management emphasized the early diagnosis and targeted therapy (Aletaha and Smolen, 2018; Smolen et al., 2018) which can slow down disease progression and avoid irreversible disability. However, there is little guidance to make the detailed suggestion for pre-clinical and pre-RA patients (Fraenkel et al., 2021). To date, many trials have tried to answer the definition and management of pre-clinical RA and pre-RA (Lard et al., 2001; Nell et al., 2004; van Dongen et al., 2007; Lukas et al., 2011; Wevers-de Boer et al., 2012; van Aken et al., 2014; Stamm et al., 2018; Gerlag et al., 2019). For example, van Dongen et al. (2007) found that MTX was beneficial in postponing the diagnosis of RA (ACR 1987 criteria), and retarding radiographic joint damage in undifferentiated arthritis (UA) patients. Lukas et al. (2011) showed that early initiation of DMARD therapy reduces the radiological progression of 12 months. In all, the decision supported that pre-RA or pre-clinical RA patients benefit from early initiation of DMARD.

Prophylactic treatment is a major feature of traditional Chinese medicine (TCM). In the recent pandemic of SARS-Cov-2, the combination of Western medicine and TCM had better performance on preventing disease transformation than Western medicine only (Li et al., 2020; Wu et al., 2021). To date, there is no TCM-related trial aimed at preventing RA onset. Non-specific musculoskeletal (MSK) symptoms with positive anticyclic citrullinated peptide (anti-CCP) antibodies patients have a high risk of developing into RA in 12 months (Rakieh et al., 2015). Besides, some anti-CCP negative arthralgia patients also have a high risk of developing RA (van Steenbergen et al., 2017). Thus, we would conduct a multi-center, double-blind, placebo-controlled randomized trial recruiting both anti-CCP positive and negative patients, to demonstrate the efficacy and safety of Yunpi Qufeng Chushi formula (YQCF) in preventing the development of RA.

## 2 Materials and Methods

### 2.1 Study Setting

The multi-center, double-blind, placebo-controlled clinical trial is conducted in 13 hospitals nationwide including First Affiliated Hospital of Zhejiang Chinese Medical University, Second Affiliated Hospital of Zhejiang Chinese Medical University, Third Affiliated Hospital of Zhejiang Chinese Medical University, Xiyuan Hospital of China Academy of Chinese Medical Sciences, Guanganmen Hospital of China Academy of Chinese Medical Sciences, Dongfang Hospital of Beijing University of Chinese Medicine, China-Japan Friendship Hospital, Shanghai Hospital of Traditional Chinese Medicine, First Affiliated Hospital of Zhejiang University School of Medicine, Second Affiliated Hospital of Zhejiang University School of Medicine, Sir Run Shaw Hospital of Zhejiang University School of Medicine, First Affiliated Hospital of Guangzhou University of Traditional Chinese Medicine, and First Affiliated Hospital of Anhui University of Chinese Medicine.

### 2.2 Participants

A total of 390 participants aged 18–70 will be recruited. Inclusion and exclusion criteria are presented as follows.

To date, the definition (or criteria) of pre-RA is controversial. We propose our definition based on the trials mentioned above. For the patients with positive anti-CCP antibodies: at least 1 joint involvement (any swollen or tender joint on examination) and do not meet any RA classification criteria [ACR 1987 revised criteria (Arnett et al., 1988) and ACR/EULAR 2010 criteria (Aletaha et al., 2010)]. For the patients with negative anti-CCP antibodies: meet with at least 5 parameters according to the EULAR definition of arthralgia suspicious for progression to RA (van Steenbergen et al., 2017) (details available in Table 1).

**TABLE 1:** EULAR defined characteristics describing arthralgia at risk for RA (adapted from van Steenbergen et al., 2017).

History taking
1. Joint symptoms of recent onset (duration <1 year)
2. Symptoms located in MCP joints
3. Duration of morning stiffness ≥60 min
4. Most severe symptoms present in the early morning
5. Presence of a first-degree relative with RA
Physical examination:
6. Difficulty with making a fist
7. Positive squeeze test of MCP joints

### 2.3 Inclusion Criteria

(1) Meet the definition which we proposed of pre-RA.
(2) The course of disease is between 6 weeks and 6 months.
(3) Meet with the diagnostic criteria of TCM syndrome of spleen deficiency and wind-damp blockade syndrome (available in Table 2).
(4) The men and women patients are aged 18–70 years.
(5) Patients provided with informed consent and signed agreement.

**TABLE 2:** Diagnostic criteria of spleen deficiency and wind-damp blockade syndrome.

Primary symptoms and signs	(1) Joint stiffness
	(2) Joint wandering pain
	(3) >1 joint pain
	(4) Epigastric distention
Secondary symptoms and signs	(1) Heavy body and limbs
	(2) Loose stool
	(3) Loss of appetite
	(4) Tongue condition: pale tongue with white coating
	(5) Pulse condition: thin and soft pulse

Patients with four primary symptoms or three primary symptoms and two or more secondary symptoms could be diagnosed as spleen deficiency and wind-damp blockade syndrome.

### 2.4 Exclusion Criteria

(1) Any DMARDs, including conventional synthetic DMARDs (csDMARDs), targeted synthetic DMARDs (tsDMARDs), and biological DMARDs (bDMARDs), have been used for treatment within 2 months before enrollment. Further explanation: a patient is undertaking some DMARDs and is willing to take part in the study, they can stop the DMARDs for 2 months and subsequently recheck the necessary examination to take part in the trial.
(2) Patients have recently taken glucocorticoids, such as prednisone.
(3) With a history of severe organ diseases or mental diseases.
(4) Patients are allergic to the drugs involved in the study protocol or have contraindications.

### 2.5 Interventions

Subjects will be randomly allocated to either the intervention group or the control group through a central randomization system. Treatment will continue for 48 weeks. The visit time will be set at 0 weeks (recruitment), 12, 24, and 48 weeks. YQCF granules and the placebo granules were manufactured by China Resources Sanjiu Pharmaceutical Co., Ltd. One day dosage of YQCF granules: Atractylodis rhizoma [Asteraceae; Atractylodes lancea (Thunb.) DC.]: 2.5 g (equally 15 g herb medicine, abbreviated as 15 g), Aaposhnikoviae radix [Apiaceae; Saposhnikovia divaricata (Turcz. ex Ledeb.) Schischk.]: 0.3 g (6 g), Schizonepetae herba [Lamiaceae; Nepeta tenuifolia Benth.]: 1.2 g (12 g), Sinomenii caulis [Menispermaceae; Sinomenium acutum (Thunb.) Rehder and E.H.Wilson]: 2 g (15 g), Lonicerae japonicae flos [Caprifoliaceae; *Lonicera japonica* Thunb.]: 3 g (15 g), Smilacis glabrae rhizoma [Smilacaceae; Smilax glabra Roxb.]: 1.33 g (20 g), Cynanchi paniculati radix et rhizoma [Apocynaceae; Vincetoxicum mukdenense Kitag.]: 2 g (12 g), Coicis semen [Poaceae; Coix lacryma-jobi var. ma-yuen (Rom. Caill.) Stapf]: 1.5 g (15 g), and Glycyrrhizae radix et rhizoma [Fabaceae; Glycyrrhiza glabra L.]: 2 g (6 g). Drug compatibility: 1) joint significantly swelling and pain: Leigongteng [Celastraceae; Tripterygium wilfordii Hook. f.]: 1.5 g (15 g), cuscutae semen [Convolvulaceae; Cuscuta chinensis Lam.]: 0.75 g (15 g), Polygoni cuspidati rhizoma et radix [Polygonaceae; Reynoutria japonica Houtt.]: 1 g (15 g); 2) exuberant heat: Anemarrhenae rhizoma [Asparagaceae; Anemarrhena asphodeloides Bunge]: 0.75 g (15 g); 3) exuberant cold: Cinnamomi ramulus [Lauraceae; Neolitsea cassia (L.) Kosterm.]: 1.25 g (15 g); 4) low back pain: Eucommiae cortex [Eucommiaceae; Eucommia ulmoides Oliv.]: 0.6 g (12 g); 5) pain above the waist: Mori ramulus [Apiaceae; Hansenia weberbaueriana (Fedde ex H. Wolff) Pimenov and Kljuykov]: 0.9 g (18 g); 6) pain below the waist: Cyathulae radix [Apiaceae; Angelica biserrata (R.H. Shan and C.Q. Yuan) C.Q. Yuan and R.H. Shan]: 1.49 g (10 g). The YQCF granules and placebo will be packed into identical-looking medicine bags. Dosage: two bags per day. Our research group published a study on the identification of the main components of YQCF by liquid chromatography–mass spectrometry. The methods and the results were available at Li et al. (2021). Besides, we also applied quality control using HPLC, the methods and the results are available in Supplementary Material S5. The quality control data provided by China Resources Sanjiu Pharmaceutical Co., Ltd. are available in Supplementary Material S6.

Nonsteroidal anti-inflammatory drugs (NSAIDs) will be allowed to be prescribed when the patients suffer from pain. DAMARs and glucocorticoids will not be allowed in the study. The untaken drug will be returned.

Mild and moderate adverse events (AEs) will change the composition of YQCF granules according to the relative granules AEs. For example, Sinomenium acutum (Thunb.) Rehder and E.H. Wilson will be removed because it would cause cutaneous pruritus. Severe AEs will stop the intervention and the patient will withdraw.

### 2.6 Outcomes

#### 2.6.1 Primary Outcomes

##### 2.6.1.1 RA Transition Rate

Definition: pre-RA patients who fulfill any criteria of RA classification at any time will be defined as “RA transition.” Survival time will be recorded as the number of days between the date of informed consent and the date of transition.

#### 2.6.2 Secondary Outcomes

1. Changes in visual analogue scale (VAS): Measurement will be performed at every visit.
2. Changes in patient’s global assessment of disease activity (PGA) and physician’s global assessment of disease activity (MDGA): Measurement will be performed at every visit.
3. Changes in clinical disease activity index (CDAI) and simplified disease activity index (SDAI): Measurement will be performed at every visit. The evaluation methods were described carefully in a former study (Aletaha and Smolen, 2005).
4. Changes in disease activity score 28 (DAS28): Measurement will be performed at every visit. The evaluation methods were described carefully in a former study (Prevoo et al., 1995).
5. Changes in TCM syndromes efficacy score scale: TCM syndromes efficacy score ranges from from 0 to 45. Measurement will be performed at every visit.
6. Changes in Health Assessment Questionnaire-Disability Index (HAQ-DI): Measurement will be performed at baseline and week 48. The evaluation methods are available at Khanna et al. (2005).
7. Changes in FACIT-Fatigue score scale: Measurement will be performed at baseline and week 48. The evaluation methods are available in the Yellen et al. (1997) study.
8. Changes in color Doppler ultrasound semi-quantitative score of joints. Measurement will be performed at baseline and week 48. We consider an ultrasound score based on 7 joints of the clinically dominant hand and foot evaluation methods (Backhaus et al., 2009).
9. Changes in CRP, ESR, RF, and anti-CCP. CRP and ESR will be performed at every visit. RF and anti-CCP will be performed at baseline and week 48.

#### 2.6.3 Safety Outcomes

AEs will be monitored for 48  weeks from the time when the subject signed the informed consent and enrolled in the trial to the last follow-up. Especially, visit 2 (4 weeks ± 2 days) is set to monitor the AEs. The description of severity (grades) and system organ class (SOC) of AEs will follow the Common Terminology Criteria for Adverse Events (CTCAE) Version 5.0 (SERVICES, 2017). The AEs would be coded using the current version of the Medical Dictionary for Regulatory Activities (MedDRA). The relationship between AEs and intervention will be judged according to the adverse drug reaction report and testing manual published by the Department of Drug Safety Supervision, State Food and Drug Administration of the People’s Republic of China (Guan et al., 2008).

### 2.7 Participant Timeline

**FIGURE 1:**
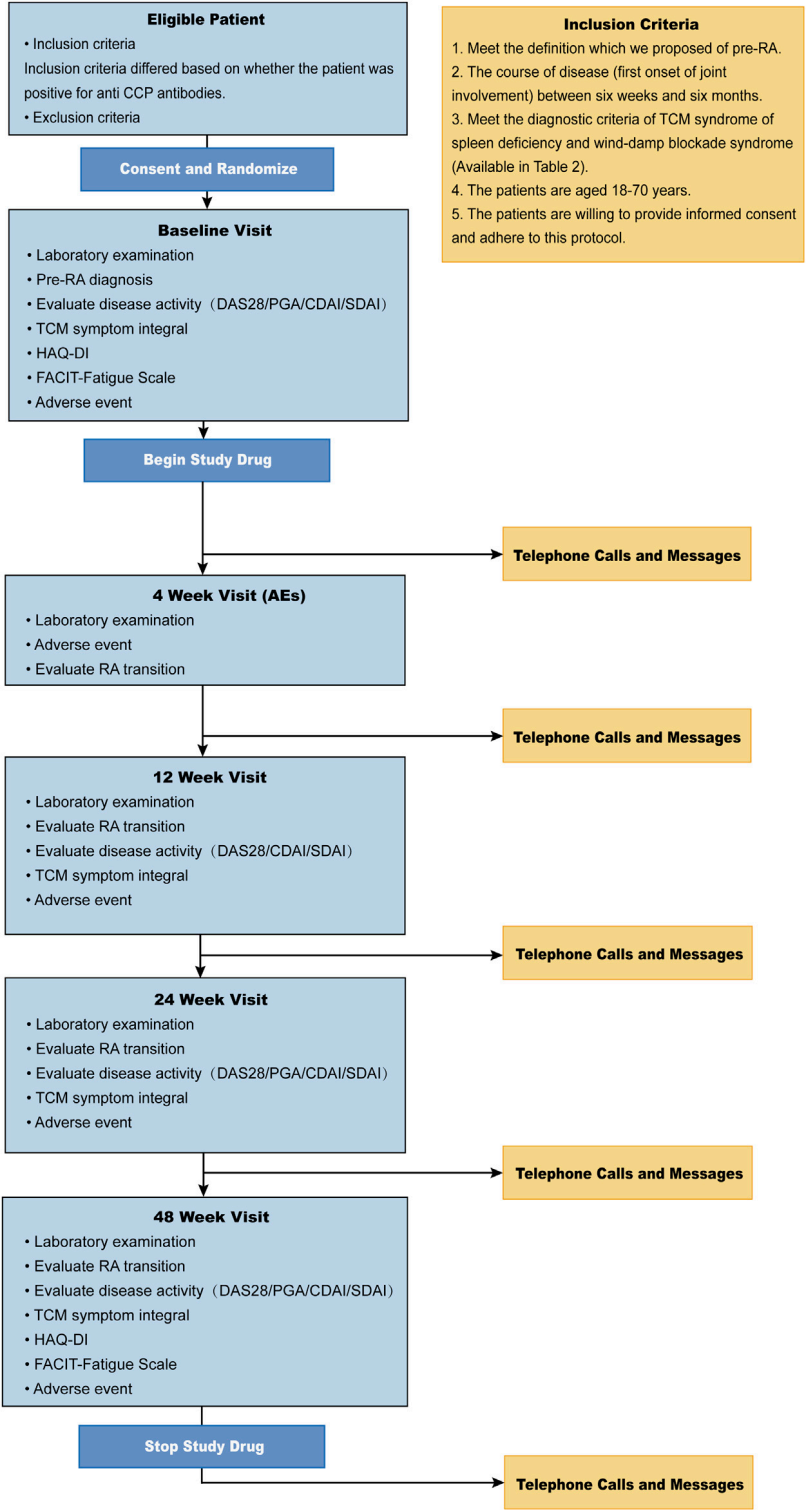
The flow diagram of the study.

The study will last 3 years: from July 2019 to July 2022. The timeline and study procedure are available in Table 3 and Figure 1.

**TABLE 3:** Study procedure table.

Visit Project	Screen period (baseline)		Visit			
	1		2	3	4	5
Time of visit	−4 weeks-0 days	0 ± 2 days	4 weeks ± 2 days	12 weeks ± 3 days	24 weeks ± 5 days	48 weeks ± 5 days
General information	●					
Family history	●					
Medical history	●					
Drug allergy history	●					
Pre-trial medication*	●					
Vital signs	●		●	●	●	●
Physical examination	●			●	●	●
Laboratory examination						
Routine blood test	●		●	●	●	●
CRP	●			●	●	●
ESR	●			●	●	●
Routine urine/stool test	●			●	●	●
RF + ASO	●			●	●	●
Blood biochemical examination**	●		●	●	●	●
ACPA	●					●
Color Doppler ultrasound		●		●	●	●
Other laboratory results		●		●	●	●
Pre-RA diagnosis	●					
Evaluate RA conversion			●	●	●	●
TCM syndrome diagnosis	●					
Inclusion/exclusion criteria	●					
Screening results	●					
Sign informed consent form	●					
Evaluate disease activity (DAS28/PGA/CDAI/SDAI/VAS)		●		●	●	●
TCM symptom integral		●		●	●	●
Health Assessment Questionnaire-Disability Index		●				●
FACIT-Fatigue Scale		●				●
Suspension standards			●	●	●	●
Prescription		●	●	●	●	●
Combined drugs***		●	●			
Adverse event***		●	●	●	●	●
Distribution of medicine by express mail		●	●	●	●	●
End of study summary						●

*Record medications that were used and discontinued prior to the study. Drugs that were not discontinued prior to the study and entered the treatment period should be recorded in “Combined Drugs”. **Only liver function needs to be tested at 2 weeks. ***Observe continuously throughout the study and record when it occurs.

### 2.8 Sample Size

Based on the primary outcome, we use the model of “tests for two proportions” in PASS 15.0. Alternative hypothesis: two-sided. Test type: Z-test (unpooled). Power was set at 0.9 and alpha was set at 0.05. The intervention group and control group were allocated a 1:1 ratio. The effect size of the treatment group (P1): based on the results of our former small sample of observational study (results unpublished), about 53.26% of patients would transit to RA after 1 year of TCM treatment. The effect size of the control group (P2): without any medication interventions, about 70% of early-stage UA patients with ACPAs meet the 1987 ACR criteria 1-year follow-up (Firestein et al., 2016). We would need 173 subjects in each group. Predicting a 10% dropout rate, the subjects rise to 385. Considering there were 13 centers that took part in the studies, the final subject was set at 390 (30 subjects per center).

### 2.9 Recruitment

For achieving adequate participant enrolment: 1) We placed the recruitment advertisement in the hospital outpatient waiting hall; 2) we published the announcement in a WeChat individual official account (one of the most popular social networking software in China); and 3) we referred patients who meet inclusion criteria in cooperation with primary care units.

### 2.10 Allocation, Sequence Generation, Blinding, and Implementation

Drug clinical trial data management system [abbreviated as electronic case report form (eCRF) system)] developed by Nanjing Haitai Medical Information System Co., Ltd. is a secure web application for building and managing online surveys and databases. Sequence generation: a multicenter block random method was used. The random number of multicenter groups was generated through the corresponding randomization program of SAS software (version 9.4). The relevant parameters and results of the SAS randomization program were saved by Zhejiang Chinese Medical University (study design unit). The sequence was uploaded to the eCRF system and researchers took part in the study retrieve sequence for recruiting patients. Zhijun Xie (one of the designers), outcome assessors, and workers in the drug manufacturing center are not masking (participants and investigators are blinding). In the real practice of TCM diagnosis and treatment, TCM doctors usually prescribe 14-d to 28-d treatment based on the patients’ symptoms and TCM patents. Thus, the researchers would upload the prescriptions and patient express information to Zhijun Xie for simulating a real TCM situation. Subsequently, Zhijun Xie would upload patient express information and which drug (placebo or YQCF, including drug compatibility) the manufacture center will process. The product would be delivered to the patients directly based on their express information. All the participants who save the patient personal privacy information strictly abide by the confidentiality regulations.

### 2.11 Data Collection Methods and Management

Data will be collected in the paper and electronic CRF via the clinic and the Internet (using the eCRF system), respectively. Paper CRF will be delivered to clinical centers. Training and support for using the eCRF system will be made available to researchers by the Zhejiang Chinese Medical University. For promoting participant retention and complete follow-up, first-line investigators will inform the patients when they should go to the hospital to complete follow-up via telephone and WeChat. Two independent investigators will check the data values.

### 2.12 Statistical Methods

#### 2.12.1 Primary Outcome

The final RA transition rate will be performed by Pearson’s chi-square or Fisher’s exact chi-square tests. In addition, RA transition survival analysis will be performed by Kaplan-Meier survival analysis. Binary logistic regression is utilized to investigate the multiple variables which can affect RA transition rate. The variables include sex, age, anti-ccp and RF state, family history, DAS28, continuous NSAIDs use, and YQCF use. Per-protocol (PP) analysis and intention-to-treat (ITT) analysis will be applied in chi-square tests, survival analysis, and binary logistic regression.

#### 2.12.2 Secondary Outcome

All the secondary outcomes will be summarized in Mean ± SD and analyzed by repeated measurement analysis of variance.

In addition, scores of DAS28, CDAI, and SDAI will be classified as disease remission, low disease activity, moderate disease activity, and high disease activity according to the relevant definition (Prevoo et al., 1995; Aletaha and Smolen, 2005). The categorical data will be performed by chi-square tests by row sums (different disease activities) and column sums (groups).

#### 2.12.3 Safety Outcome

Safety outcomes in ITT data will be summarized according to the different SOC with CTCAE grades. Every category of AEs will be performed by chi-square tests by row sums (different CTCAE grades) and column sums (groups). In addition, we would screen out the YQCF-unrelated AEs to validate the safety of YQCF. The data will be summarized and analyzed as the methods mentioned above.

#### 2.12.4 Subgroup Analysis

The subgroup analysis of the primary outcome and the secondary outcome will be based on anti-CCP positive or negative.

### 2.13 Data Monitoring, Harms, and Auditing

Zhejiang Chinese Medical University led a steering committee and data monitoring committee (DMC) which is composed of experienced clinical researchers and academic investigators in this field. The committee will carry every month online and half a year of on-site auditing. The committee will oversee the data and safety of the study and will conduct primary outcome interim analyses using Lan-DeMets with O'Brien Fleming to oversee the efficacy and safety when it reaches 50% of the recruitment. Moreover, the committee would assess the status and quality of the study, such as the rate of recruitment and withdrawal. Overall, the YGCG is relatively safe, and the AEs are mild. Every patient and their family members could report any AEs/conditions to the investigators via WeChat and the investigators would immediately take action.

## 3 Ethics and Dissemination

This trial has been approved by the Zhejiang Chinese Medical University Ethics Committee under number 2019-045. Approval has been obtained from the local institutional review boards at all participating centers. The results of RCT will be published in peer-reviewed journals. All subjects were required to obtain permission to publish the results of the study and to ensure anonymity and confidentiality. All data will be processed under the rules of the government and law. All researchers will guarantee the anonymity of patients and will not disclose the names of patients in forms, reports, or articles unless required by law. Only authorized individuals can obtain patient information.

## 4 Discussion

Nowadays, more and more evidence points out the importance of early diagnosis and treatment of RA (Combe et al., 2017; Smolen et al., 2020). However, there is a lack of treatment schemes for pre-RA patients. The trial is the first multi-center, double-blind, placebo-controlled RCT carried out in China to evaluate the TCM in stopping the pre-RA progression. It will provide evidence of TCM in disease prevention of RA. In this study, we also focus on health-related assessment (such as HAQ-DI and FACIT-Fatigue) and ultrasound changes in pre-RA patients. Based on the trial, we will conduct an observational study to evaluate the economic benefit, efficacy, and safety of our treatment scheme as well.

In this study, we propose our definition of pre-RA. Studies have shown that anti-CCP antibody-positive makes a great contribution to the development of RA and many of the arthralgia individuals with anti-CCP antibody-positive would progress to RA in the first year (Rantapää-Dahlqvist et al., 2003; van Steenbergen et al., 2017). Anti-CCP antibody-negative individuals were likely to progress to other rheumatism diseases instead of RA (Hiura et al., 2013). However, anti-CCP antibody-negative individuals who meet the EULAR definition have a high risk of developing RA (van Steenbergen et al., 2017). Thus, we recruit anti-CCP antibody positive and negative individuals to provide accurate evidence in the treatment of pre-RA. Moreover, anti-CCP antibody status would affect the efficacy of DMARDs in pre-RA. For example, methotrexate could not stop the anti-CCP-negative individuals to progress to RA (van Aken et al., 2014). TCM may prevent pre-RA progression by targeting multiple molecules regardless of the anti-CCP antibody status.

In this study, the following measures have been taken for diagnosis and differential diagnosis of pre-RA: according to the RA classification criteria, joint involvement, up to 5 scores and only 1 point away from the RA diagnostic threshold, is defined as: any swollen or tender joint on examination, which may be confirmed by imaging evidence of synovitis (Aletaha et al., 2010). The exact joint involvement assessment is critical in pre-RA diagnosis. Both ultrasound and MRI are effective techniques to evaluate joint inflammation. However, in view of the advantages of low cost and convenience, ultrasound may be a better choice in pre-RA diagnosis (Xu et al., 2017). Other anti-nuclear antibodies (ANA) also would be detected at baseline for diagnosing other rheumatoid diseases.

This study also has certain limitations. The sample size and trial duration are calculated based on the anti-CCP-positive individuals. Anti-CCP level not only affects the efficacy of DMARDs but also is related to the onset time of arthritis (Burgers et al., 2017). Anti-CCP-positive individuals are more likely to develop arthritis and RA as compared to anti-CCP-negative individuals. More subjects and longer follow-up visits are needed in prognosis analysis. The guidelines published by ACR and EULAR stated that an early treat-to-target (T2T) strategy will improve outcomes, such as bone erosions (Smolen et al., 2020; Fraenkel et al., 2021). However, due to the limited observation time, we cannot answer whether the use of TCM schemes can achieve better outcomes.
