# Supplementary material for: Yunpi Qufeng Chushi Formula for Pre-Rheumatoid Arthritis: Study Protocol for a Multiple-Center, Double-Blind, Placebo-Controlled Randomized Controlled Trial
Source: Front Pharmacol. 2022 Feb 14;13:793394. doi: 10.3389/fphar.2022.793394 (PMC8882904; doi:10.3389/fphar.2022.793394)
Supplement: Supplementary file 1 [file DataSheet1.zip › Supplementary material 1 Ethics approval.pdf.pdf]

浙江中医药大学医学伦理委员会

伦理审查批件

批件号：2019-045

审查会议日期	2019 年 11 月 13 日		
审查会议地点	浙江省杭州市滨江区滨文路 548 号浙江中医药大学		
临床研究批文	国家重点研发计划项目(2018YFC1705501)		
临床研究项目	RA 前状态中医防治方案的循证评价研究（运脾祛风除湿颗粒防治 RA 前状态随机双盲研究）		
审查文件	1、 科研项目修正申请报告<br>2、 临床研究方案（版本号：201910-1）<br>3、 临床研究手册（版本号：201910-1）		
申办者	温成平	临床研究主要负责单位	浙江中医药大学
主要研究者	温成平、谢志军、范永升、曹炜、吴华香、高祥福、林昌松、陶庆文、许凤全、鲁科达、王新昌		
伦理审查方式	■ 会议审查　　　□ 加快审查		
投票结果	委员人数 17 人，本次会议出席委员 17 人，参加投票委员 17 人，同意 17 票，修改后同意 0 票，修改后重审 0 票，不同意 0 票，终止或暂停已批准的试验 0 票。		
审查意见	审查结果：■同意　　　□不同意<br>根据卫生部《涉及人的生物医学研究伦理审查办法（试行）》(2007)、CFDA《药物临床试验质量管理规范（2003)》、《医疗器械临床试验规定（2004)》、WMA《赫尔辛基宣言》和 CIOMS《人体生物医学研究国际道德指南》的伦理原则，经本伦理委员会审查，同意按所批准的临床研究方案、知情同意书、招募材料开展本项研究。请遵循 GCP 原则、遵循伦理委委员会批准的方案开展临床研究，保护受试者的健康与权力。<br>◆ 研究开始前，请申请人完成临床试验注册。<br>◆ 研究过程中若变更主要研究者，对临床方案、知情同意书、招募材料等的任何修改，请申请人提交修正案审查申请。<br>◆ 发生严重不良事件(SAE)，请申请人及时提交 SAE 报告，本伦理委员会有权根据 SAE 做出新的审查决定；提交报告之后，尽快提交详细的严重不良事件随访报告。<br>◆ 请按照伦理委员会规定的持续审查频率，申请人在截止日期前 1 个月提交持续审查申请报告，本伦理委员会有权根据实际开展情况修改持续审查频率，并有权根据实际审查情况做出新的决定；当出现任何可能显著影响实验进行、或增加受试者危险的情况时，请申请人及时向伦理委员会提交书面报告。<br>◆ 研究纳入了不符合纳入标准或符合排除标准的受试者，符合终止试验规定而未让受试者退出研究，给予错误治疗或剂量，给予方案禁止的合并用药等没有遵从方案的情况；或可能对受试者的权益/健康、以及研究的科学性造成不良影响等违背 GCP 原则的情况，请申办方/监察员/研究者提交违背方案报告。<br>◆ 申请人暂停或提前终止临床研究，请及时提交暂停/终止研究报告。<br>◆ 完成临床研究，请申请人提交结题报告。<br>◆ 及时书面报告中心伦理的重要决定。<br>◆ 凡涉及中国人类已遗传资源、需要报批的研究项目，需要在获得中国人类遗传资源管理办公室批准后才能开始研究。		
有效期	2019 年 10 月 16 日～2022 年 10 月 15 日		
联系电话	0571-86613536		
主任委员签字	范永升		
浙江中医药大学医学伦理委员会（盖章）			
日期：　2019 年 11 月 15 日			

浙江中医药大学医学伦理委员会
