# Supplementary material for: Yunpi Qufeng Chushi Formula for Pre-Rheumatoid Arthritis: Study Protocol for a Multiple-Center, Double-Blind, Placebo-Controlled Randomized Controlled Trial
Source: Front Pharmacol. 2022 Feb 14;13:793394. doi: 10.3389/fphar.2022.793394 (PMC8882904; doi:10.3389/fphar.2022.793394)
Supplement: Supplementary file 1 [file DataSheet1.zip › Supplementary material 2 Informed consent form (Chinese version).docx.docx]

知情同意书·知情告知页

亲爱的患者

您的医生已经确诊您患有类风湿关节炎前状态（RA前状态）疾病。

我们将邀请您参加一项RA 前状态中医防治方案的循证评价研究，并将与单纯西药治疗进行比较，以观察他们对于RA前状态的疗效和安全性。

在您决定是否参加这项研究之前，请尽可能仔细阅读以下内容，它可以帮助您了解该项研究以及为何要进行这项研究，研究的程序和期限，参加研究后可能给您带来的益处、风险和不适。如果您愿意，您也可以和您的亲属、朋友一起讨论，或者请您的医生给予解释，帮助您做出决定。

研究介绍

　　一、研究背景和研究目的

类风湿性关节炎（rheumatoid arthritis，RA）是一种难治性的全身性免疫风湿病。RA前状态（ACPA阳性的未分化关节炎）1 年的RA 转化率为70%。目前西药对防止RA前状态进展为RA缺乏基于循证证据的治疗方案，而中医药在前期预防RA前状态转化为RA、协同治疗RA活动期的增效减毒作用方面均有优势，但存在高质量循证证据缺乏、作用机制不够明确和难以推广应用等重要问题。为发挥RA中医分期治疗方案的优势，“未病先防”，早期诊疗，有效降低RA发生率，故进行本研究

本研究的目的是揭示运脾祛风除湿颗粒预防RA 前状态转化为RA 的临床疗效（降低RA 转化率、改善关节炎等）和安全性，形成高质量临床证据，形成有疗效优势的预防RA 前状态转化的中医药方案。本研究是结合母方案设计，多中心前瞻性随机双盲对照试验。将在在全国13家三级甲等综合性医院开展，包括浙江中医药大学附属第一、二、三医院、中国中医科学院广安门医院、西苑医院、北京中医药大学东方医院、中日友好医院、上海市中医医院、浙江大学医学院附属第二、第一及邵逸夫医院、广州中医药大学第一临床医学院、安徽中医药大学第一附属医院，共计390例RA 前状态患者。研究方法和程序：首先对患者病情进行评估，符合入选标准的患者将随机进入不同的组别。分组如下：

对照组：运脾祛风除湿颗粒安慰剂+对症治疗

中药组：运脾祛风除湿颗粒+对症治疗

本项研究已经得到国家科技部批准。浙江中医药大学伦理委员会已经审议此项研究是遵从赫尔辛基宣言原则，符合医疗道德的。

　　二、哪些人不宜参加研究

①2个月内使用了DMARDs治疗者。

②应用糖皮质激素治疗者。

③有严重脏器病变者或精神类疾病者。

④对研究方案中涉及的药物过敏或有使用禁忌者。

　　三、如果参加研究将需要做什么

1. 在您入选研究前，您将接受以下检查以确定您是否可以参加研究：

医生将询问、记录您的病史，对您进行体格检查。

您需要做血常规、血沉、尿常规、类风湿因子、血生化、粪潜血、关节核磁共振、关节彩色超声、抗CCP抗体及其它相关实验室及辅助检查。

2. 若您以上检查合格，将按以下步骤进行研究（按随访时点详细陈述治疗及各检查项目）

研究开始将根据计算机提供的随机数字，决定您接受中药方案或西药方案。参加这项研究的患者分别有50%的可能性被分入这两个不同的组别。您和您的医生都无法事先知道和选择任何一种干预措施。治疗观察将持续48周。

入选后您将在研究第12、24、48周均应到医院就诊，医生将询问记录您病情的变化，给您做体格检查，并做血常规、血沉、C反应蛋白、尿和粪便常规、血生化、类风湿因子及其它相关实验室及辅助检查，医生将记录检测结果并开具处方，研究中将每隔2周分发药物。此外，研究第24周需要加做关节彩色超声，研究第48周需要加做关节彩色超声、关节核磁共振、抗CCP抗体。

3. 需要您配合的其他事项

您需要按医生和您约定的随访时间来医院就诊。您的随访非常重要，因为医生将判断您接受的研究措施是否真正起作用。

您需要按医生指导用药，您在每次随访时都必须归还未用完的药物及其包装，并将正在服用的其它药物带来，包括您有其它合并疾病须继续服用的药物。

在研究期间您不能使用治疗RA前状态或RA的其它中药。如您需要进行其它治疗，请事先与您的医生取得联系。要避免日晒，少吃海鲜及紫苜蓿类食物，生活节律，保持心情舒畅，避免过劳。

四、参加研究可能的受益

您和社会将可能从本项研究中受益。此种受益包括您的病情有可能获得改善，以及本项研究可能帮助开发出一种新治疗方法，以用于患有相似病情的其他病人。

您将在研究期间获得良好的医疗服务，享受优先挂号、免费咨询。

五、参加研究可能的不良反应、风险和不适、不方便

中药的副作用：部分患者服用后可见胃肠道不适反应等。

尽管到目前为止没有发现该研究方法有任何不良反应，如果在研究中您出现任何不适，或病情发生新的变化，或任何意外情况，不管是否与药物有关，均应及时通知您的医生，他/她将对此作出判断和医疗处理。

医生和课题组将尽全力预防和治疗由于本研究可能带来的伤害。如果在临床试验中出现不良事件，医学专家委员会将会鉴定其是否与试验药物有关。申办者将对与试验相关的损害提供治疗的费用及相应的经济补偿，这一点已经在我国《药物临床试验质量管理规范》中作出了规定。

您在研究期间需要按时到医院随访，做一些理化检查，这些都可能给您造成麻烦或带来不方便。

此外，（研究干预）可能出现无效的情况，以及因治疗无效或者因合并其他疾病等原因而导致病情继续发展。在研究期间，如果医生发现本项研究所采取的（研究干预）措施无效，将会中止研究，改用其他可能有效的治疗措施。

六、有关费用

如果发生与试验相关的损害，申办者将支付您的医疗费用。如果因严重不良反应住院医疗，申办者还将提供适当的营养费、误工的工资和奖金的补偿费。

如果您同时合并其他疾病所需的治疗和检查，将不在免费的范围之内。

七、个人信息是保密的吗？

您的医疗记录（研究病历/CRF、化验单等）将完整地保存在医院，医生会将化验检查结果记录在您的门诊病历上。研究者、申办者代表、伦理委员会和药品监督管理部门将被允许查阅您的医疗记录。任何有关本项研究结果的公开报告将不会披露您的个人身份。我们将在法律允许的范围内，尽一切努力保护您个人医疗资料的隐私。

除本研究以外，有可能在今后的其他研究中会再次利用您的医疗记录和病理检查标本。您现在也可以声明拒绝除本研究外的其他研究利用您的医疗记录和病理标本。

八、怎样获得更多的信息？

您可以在任何时间提出有关本项研究的任何问题。您的医生将给您留下他/她的电话号码以便能回答您的问题。

如果您对参加研究有任何抱怨，请联系伦理委员会办公室。

如果在研究过程中有任何重要的新信息，可能影响您继续参加研究的意愿时，您的医生将会及时通知您。

九、可以自愿选择参加研究和中途退出研究

是否参加研究完全取决于您的自愿。您可以拒绝参加此项研究，或在研究过程中的任何时间退出本研究，这都不会影响您和医生间的关系，都不会影响对您的医疗或有其他方面利益的损失。

您的医生或研究者出于对您的最大利益考虑，可能会随时中止您参加本项研究。

您可以不参加本项研究，或中途选择退出研究。

如果您因为任何原因从研究中退出，您可能被询问有关您使用试验药物的情况。如果医生认为需要，您也可能被要求进行实验室检查和体格检查。这对保护您的健康十分有利。

十、现在该做什么？

是否参加本项研究由您自己决定。您可以和您的家人或者朋友讨论后再做出决定。

在您做出参加研究的决定前，请尽可能向您的医生询问有关问题，直至您对本项研究完全理解。

感谢您阅读以上材料。如果您决定参加本项研究，请告诉您的医生或研究助理，他/她会为您安排一切有关研究的事务。请您保留这份资料。

如果你对于此项目有疑问，可以咨询伦理委员会，办公室联系电话：0571-86613693

知情同意书·同意签字页

临床研究项目名称：RA 前状态中医防治方案的循证评价研究

申办者：浙江中医药大学

伦理审查批件号： 2019-045

同意声明

我已经阅读了上述有关本研究的介绍，而且有机会就此项研究与医生讨论并提出问题。我提出的所有问题都得到了满意的答复。

我知道参加本研究可能产生的风险和受益。我知晓参加研究是自愿的，我确认已有充足时间对此进行考虑，而且明白：

- 我可以随时向医生咨询更多的信息。
- 我可以随时退出本研究，而不会受到歧视或报复，医疗待遇与权益不会受到影响。

我同样清楚，如果我中途退出研究，特别是由于药物的原因使我退出研究时，我若将病情变化告诉医生，完成相应的体格检查和理化检查，这将对我本人和整个研究十分有利。

如果因病情变化我需要采取任何其他的药物治疗，我会在事先征求医生的意见，或在事后如实告诉医生。

我同意伦理委员会或申办者代表及研究质量监察人员查阅我的研究资料。

我同意□ 或拒绝□ 除本研究以外的其他研究利用我的医疗记录和病理检查标本。

我将获得一份经过签名并注明日期的知情同意书副本。

最后，我决定同意参加本项研究。

患者签名： 　＿ ＿ ＿ ＿ 年 ＿ ＿ 月 ＿ ＿ 日

患者联系电话： 手机号：

———————————————————————————————————————

我确认已向患者解释了本试验的详细情况，包括其权利以及可能的受益和风险，并给其一份签署过的知情同意书副本。

医生签名： 日期：＿ ＿ ＿ ＿ 年 ＿ ＿ 月 ＿ ＿ 日

医生的工作电话： 手机号：

———————————————————————————————————————

伦理委员会办公室联系电话：0571-86613536
