# Supplementary material for: Yunpi Qufeng Chushi Formula for Pre-Rheumatoid Arthritis: Study Protocol for a Multiple-Center, Double-Blind, Placebo-Controlled Randomized Controlled Trial
Source: Front Pharmacol. 2022 Feb 14;13:793394. doi: 10.3389/fphar.2022.793394 (PMC8882904; doi:10.3389/fphar.2022.793394)
Supplement: Supplementary file 1 [file DataSheet1.zip › Supplementary material 3 Informed consent form (English version).docx.docx]

**In****formed consent form (Informed page)**

Dear patient,

Your doctor has confirmed that you are suffering from pre-rheumatoid arthritis (pre-RA).

We will invite you to participate in an evidence-based evaluation study of traditional Chinese Traditional Medicine (TCM) prevention and treatment plan for pre-RA, and compare it with western medicine treatment to observe their efficacy and safety for pre-RA.

Before you decide whether to participate in the study, please read the following contents as carefully as possible, which can help you understand the study, why to conduct the study, the procedure and duration of the study, and the benefits, risks, and discomfort that may be brought to you after participating in the study. If you like, you can also discuss it with your relatives and friends, or ask your doctor to explain and help you make a decision.

**Study introduction**

**Ⅰ. Research background and research purpose.**

RA is a refractory systemic immune rheumatism. The 1-year RA conversion rate of pre-RA (ACPA positive undifferentiated arthritis) was 70%. At present, western medicine lacks an evidence-based treatment scheme to prevent the pre-RA state from progressing to RA, while traditional Chinese medicine has advantages in preventing the pre-RA state from transforming to RA in the early stage and synergistic and detoxification effect in the active period of RA, but there are important problems such as lack of high-quality evidence-based evidence, unclear action mechanism and difficult to popularize and apply. To give play to the advantages of the TCM phased treatment scheme of RA, "prevention before disease", early diagnosis and treatment, and effectively reduce the incidence of RA, this study was carried out.

The purpose of this study is to reveal the clinical efficacy (reduce the RA transition rate) and safety of Yunpi Qufeng Chushi Formula (YQCF) in preventing the progression of pre-RA to RA. To form high-quality clinical evidence, and form a traditional Chinese medicine scheme with curative advantage to prevent the transformation of pre-RA finally. This study is a multicenter prospective randomized double-blind controlled trial combined with the mother scheme design. 13 Grade 3A Hospitals are participating in the study, including the First Affiliated Hospital of Zhejiang Chinese Medical University, Second Affiliated Hospital of Zhejiang Chinese Medical University, Third Affiliated Hospital of Zhejiang Chinese Medical University, Xiyuan Hospital of China Academy of Chinese Medical Sciences, Guanganmen Hospital of China Academy of Chinese Medical Sciences, Dongfang Hospital of Beijing University of Chinese Medicine, China-Japan Friendship Hospital, Shanghai Hospital of Traditional Chinese Medicine, First Affiliated Hospital of Zhejiang University School of Medicine, Second Affiliated Hospital of Zhejiang University School of Medicine, Sir Run Shaw Hospital of Zhejiang University School of Medicine, First Affiliated Hospital of Guangzhou University of Traditional Chinese Medicine, First Affiliated Hospital of Anhui University of Chinese Medicine..

Research methods and procedures: firstly, the patient's condition is evaluated. Patients who meet the inclusion criteria will be randomly entered into different groups. The groups are as follows:

The grouping is as follows:

- Control group: Yunpi Qufeng Chushi Formula granules placebo and symptomatic treatment (mainly nonsteroidal anti-inflammatory drug)
- Traditional Chinese medicine group: Yunpi Qufeng Chushi Formula granules and symptomatic treatment

This study has been approved by the Ministry of Science and Technology. The Ethics Committee of Zhejiang University of Traditional Chinese Medicine has considered this study following the Declaration of Helsinki and in line with medical ethics.

**Ⅱ. Who should not participate in the study?**

1. DMARDs have been used for treatment within 2 months before enrollment.

2. Patients have recently taken glucocorticoids, such as prednisone.

3. With a history of severe organ diseases or mental diseases.

4. Patients are allergic to the drugs involved in the study protocol or have contraindications.

**Ⅲ. What will you need to do if you participate in the study?**

**1. Before you are selected for the study, you will take the following examination to determine whether you can attend the study:**

The doctor will ask, record your medical history, and take a physical examination of you.

You need to do a routine blood test, erythrocyte sedimentation rates (ESR), C-reactive protein (CRP), routine urine tests, rheumatoid factors, blood biochemical examination, fecal occult blood test, Joint MRI, color Doppler ultrasound, anti-CCP antibodies, and other related laboratory examinations.

**2. If you are enrolled in the study, the following steps will be conducted:**

You will be enrolled in the intervention group or control group with a 50% chance based on the random numbers provided by the computer. Neither you nor your doctor can know or choose any of the interventions in advance. The Treatment will last for 48 weeks.

You need to go to the hospital in the 12,24 and 48 weeks. The doctor will ask about the changes in questionnaires, schedule a physical examination, and do a routine blood test, ESR, CRP, routine urine and feces test, blood biochemistry examination, rheumatoid factor, and other related laboratory and auxiliary examinations. The doctor will record the test results and prescribe, and the drug will be distributed at 2-week intervals. In addition, an additional color Doppler ultrasound is required at study week 24. An additional color Doppler ultrasound, joint nuclear magnetic resonance, and anti-CCP antibodies are required at study week 48.

**3. Other matters requiring your cooperation.**

You need to come to the hospital at the follow-up time agreed by your doctor and you. Your follow-up is important because the doctor will judge whether the treatment you received is working.

You need to follow the medication guided by your doctor, and you must return the unused drugs and their packaging at each follow-up, and bring the other drugs you are taking, including those you have to continue when you have other combined diseases.

During the study, you cannot use other Chinese medicines to treat pre-RA or RA. If you need additional treatment, please contact your doctor in advance. You should avoid sun exposure, eat less seafood and purple alfalfa, keep your mood in a good mood and avoid overstrain.

**Ⅳ. Potential benefits from participating in the study.**

Society and you will probably benefit from this study. Benefits include the potential for improvement in your health and the possibility that this study may help develop a new treatment for other patients with similar conditions.

You will receive good medical services during the study period and enjoy preferential registration and free consultation.

**Ⅴ. Potential adverse reactions, risks, discomfort, and inconvenience to participate in the study.**

Side effects of traditional Chinese medicine: gastrointestinal discomfort reactions and so on can be seen after some patients take it.

Although no adverse reactions to the study methods have been noted to date, if you experience any discomfort during the study, or a new change in your condition, or any unexpected event, whether or not related to the drug, your doctor should be promptly notified that he/she will make a judgment and medical treatment for this.

The doctor and research group will do their best to prevent and treat possible injuries due to this study. If an adverse event occurs in a clinical trial, the Board of Medical Experts will identify if it is related to the trial drug. The sponsor will provide treatment costs and corresponding economic compensation for the damage related to the trial, which has been stipulated in China's Quality Management Code of Drug Clinical Trial.

You need to come to the hospital for follow-up on time during the study, do some physicochemical tests, which can be cumbersome or inconvenient for you.

In addition, (the study intervention) may be ineffective, and the condition continues to develop due to ineffective treatment or the combination of other diseases. During the study period, if the doctor finds the (study intervention) measures taken by this study ineffective, the study will be suspended and switched to other treatments that may be effective.

**Ⅵ. Related expenses.**

If trial-related damage occurs, the applicant will pay for your medical expenses. If hospitalized for serious adverse reactions, the applicant will also provide appropriate nutrition fees, missed wages, and compensation for bonuses.

If you combine the treatment and examination for other diseases, it will not be free.

**Ⅶ. Is the personal information kept confidential?**

Your medical records (research medical records, etc.) will be kept intact in the hospital and the doctor will record the test results on your outpatient medical records. The investigator, sponsor representative, ethics committee, and drug regulator will be allowed to access your medical records. Any public report on the results of this study will not disclose your identity. We will make every effort to protect the privacy of your personal medical information as permitted by law.

In addition to this study, your medical records and pathology examination specimens may be utilized again in other future studies. You can also now declare the denial of studies other than this study utilizing your medical records and pathology specimens.

**Ⅷ. How do you get more information?**

You can raise any questions about this study at any time. Your doctor will leave you with his / her phone number to answer your questions.

If you have any complaints about attending the study, please contact the Ethics Committee Office.

Your doctor will promptly notify you if there is any important new information during the study that may affect your willingness to continue participating in the study.

**Ⅸ. You can voluntarily choose to participate in the study and withdraw from the study midway.**

Whether to participate in the study depends entirely on your voluntary nature. You may refuse to participate in this study or withdraw from this study at any time during the study, which will not affect the relationship between you and your doctor, nor the loss of your medical or other benefits. Your doctor or investigator may suspend your participation in this study at any time for your best interest.

You may not participate in this study, or opt-out of the study midway.

If you withdraw from the study for any reason, you may be asked about your use of the trial drugs. You may also be asked for a laboratory test and physical examination if deemed necessary by your doctor. This is good for protecting your health.

**Ⅹ. What should you do now?**

Whether to participate in this study is up to your discretion. You can discuss it with your family or friends before making a decision.

Before you make your decision to participate in the study, ask your doctor about related issues as much as possible until you fully understand the study.

Thank you for reading the above information. If you decide to participate in this study, please tell your doctor or research assistant that he/she will arrange for you everything about the study. Please keep this information.

If you have questions about this project, please consult the ethics committee at 0571-86613693.

**Informed consent form (consent signature page)**

**Name of clinical research project:** Evidence based evaluation of traditional Chinese medicine (TCM) treatment options in the pre-RA state

**Applicant:** Zhejiang University of Traditional Chinese Medicine

**Ethics Review Approval No.:** 2019-045

**Consent to the declaration**

I have read the above introduction on this study and have the opportunity to discuss and raise questions with physicians on this study. All the questions I have raised have been satisfactorily answered.

I know the possible risks and benefits of participation in this study. I know that participation in the study is voluntary and I confirm that there is ample time to consider this and understand:

•I can consult my doctor for more information at any time.

•I can withdraw from this study at any time without discrimination or retaliation, and medical treatment and interests will not be affected.

I am equally clear that if I quit the study, especially due to the drug if I told the doctor the changes and completed the corresponding physical and physical examination, it would be very beneficial for me and the whole study.

If I need any other medication due to a changing condition, I will ask the doctor in advance or tell the doctor truthfully after that.

I agree that the ethics committee or sponsor consulted my research materials on behalf of the study quality monitor.

I agree with □ or reject □ the study other than this study using my medical records and pathological examination specimens.

I will obtain a signed and dated copy of the informed consent form.

Finally, I decided to agree to participate in this study.

Patient signature: Date: _ _ _ _ _ _ _ _ _ _ _ _ _ _

Patient contact number: cell-phone number:_ _ _ _ _ _ _ _ _ _ _ _

I confirm the details of the trial, including its rights and possible benefits and risks, and give patients a signed copy of the signed informed consent form.

Patient signature: Date: _ _ _ _ _ _ _ _ _ _ _ _ _ _

Patient contact number: cell-phone number:_ _ _ _ _ _ _ _ _ _ _ _

Ethics committee office contact number: 0571-86613536
