# Supplementary material for: Yunpi Qufeng Chushi Formula for Pre-Rheumatoid Arthritis: Study Protocol for a Multiple-Center, Double-Blind, Placebo-Controlled Randomized Controlled Trial
Source: Front Pharmacol. 2022 Feb 14;13:793394. doi: 10.3389/fphar.2022.793394 (PMC8882904; doi:10.3389/fphar.2022.793394)
Supplement: Supplementary file 1 [file DataSheet1.zip › Supplementary material 5 QC performed by the authors.docx.docx]

**Materials and methods**

YQCF granules were manufactured by China Resources Sanjiu Pharmaceutical Co., Ltd. 10g YQCF granules were weighed and dissolved by water for further testing. Cimicifugoside ( batch No.Y03J11H117538, Shanghai Yuanye Biotechnology Co., Ltd), 5-O-methylvisammioside (batch No. Y24J11H119521, Shanghai Yuanye Biotechnology Co., Ltd), and chlorogenic acid ( batch No. Y20A11K111541, Shanghai Yuanye Biotechnology Co., Ltd) were dissolved by methanol.

The HPLC analysis of YQCF was carried out on a Waters HPLC (Waters e2695+2998). The chromatographic separation was performed using a Phenomenex ACE Excel C18-AR column(250×4.6mm,5μm) at 25 ℃, deionized water (A), and acetonitrile (B) was used as the mobile phase for analysis. The flow rate was set at 1.0 mL/min. The elution conditions were applied with a gradient program as follows: 95–84%A for 0–5 min, 84%A for 5–15 min, 84-60%A for 15–50 min, 60-0%A for 50–65 min, 0-95%A for 65–68 min, 95%A for 68–80 min. 10 μL of the sample was injected into the HPLC system for analysis and detected at 320 nm.

**Results**

The HPLC of standards and YQCF are shown in Figure 1 and Figure 2 respectively. The concentration of cimicifugoside, 5-O-methylvisammioside, and chlorogenic acid in YQCF granules are 0.79 mg/g, 2.04 mg/g, and 64.05 mg/g respectively.

Figure 1. The HPLC of mix control.

Figure 2. The HPLC fingerprints of YQCF
