# Supplementary material for: Yunpi Qufeng Chushi Formula for Pre-Rheumatoid Arthritis: Study Protocol for a Multiple-Center, Double-Blind, Placebo-Controlled Randomized Controlled Trial
Source: Front Pharmacol. 2022 Feb 14;13:793394. doi: 10.3389/fphar.2022.793394 (PMC8882904; doi:10.3389/fphar.2022.793394)
Supplement: Supplementary file 1 [file DataSheet1.zip › Supplementary material 6 QC provided by China Resources Sanjiu Pharmaceutical Co., Ltd..docx.docx]

**Eucommiae cortex**

**1. Chromatographic conditions and system suitability test**

Octadecyl silane bonded silica gel was used as filler. Methanol-water (21:79) is used as the mobile phase; the detection wavelength is 277nm. The number of theoretical plates shall not be less than 1000 according to the peak of pinoresinol diglucoside.

**2. Preparation of reference solution**

Take an appropriate amount of rosin alcohol diglucoside as the reference substance. Add methanol to make a solution containing 0.25mg per 1ml.

**3. Preparation of test solution**

Take the product under the item of loading difference, grind it into fine, take about 0.5g, accurately weigh it, put it in Soxhlet extractor, add an appropriate amount of methanol, heat and reflux for 6 hours, recover the methanol from the extract to an appropriate amount, transfer it to 10ml measuring bottle, add methanol to the scale, shake it well, filter it, and take the continuous filtrate.

**4. Determination method**

Accurately absorb the control solution and the test solution 5 μl respectively. Inject them into the liquid chromatograph for determination.

Calculated as dry product: the content of terpineol diglucoside (c32h42o16) per 1g shall not be less than 3.5mg.

Figure 1. The HPLC of terpineol diglucoside

Figure 2. The HPLC fingerprint of eucommiae cortex

**Cinnamomi ramulus**

**1. Chromatographic conditions and system suitability test**

Octadecyl silane bonded silica gel was used as filler. Acetonitrile-0.1% phosphoric acid solution (30:70) is used as the mobile phase. The detection wavelength is 285nm. The number of theoretical plates shall not be less than 2000 according to the cinnamic acid peak.

**2. Preparation of reference solution**

Take an appropriate amount of cinnamic acid reference substance, accurately weigh it, add 50% methanol to make a solution containing 40 μg per 1ml.

**3. Preparation of test solution**

Take about 0.1g of cinnamomi ramulus and place it in a conical flask with a stopper. Add 50ml of 50% methanol and conduct ultrasonic treatment (power 250W, frequency 35kHz) for 30 minutes. Replenish with 50% methanol to the weight reduction.

**4. Determination method**

Accurately absorb the control solution and the test solution 5-10 μl respectively. Inject them into the liquid chromatograph for determination.

Calculated as dry product: the cinnamic acid (C9H8O2) per 1g shall not be less than 6.6mg.


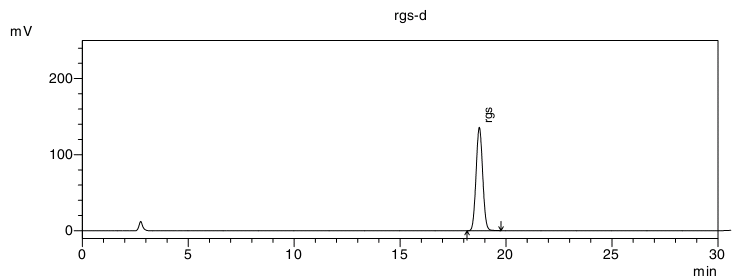


Figure 1. The HPLC of cinnamic acid


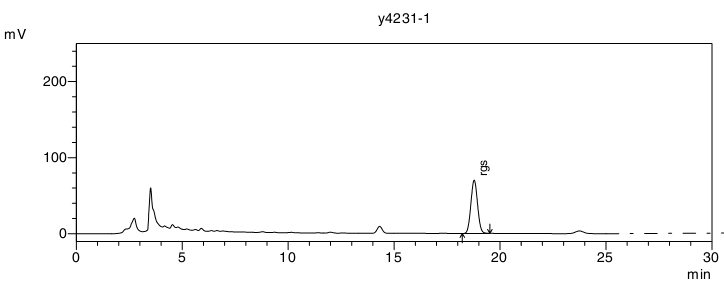


Figure 2. The HPLC fingerprint of cinnamomi ramulus

**Polygoni cuspidati rhizoma et radix**

**1. Chromatographic conditions and system suitability test**

Octadecyl silane bonded silica gel was used as filler. Methanol-0.1% phosphoric acid solution (80:20) is used as the mobile phase. The detection wavelength is 254 nm. The number of theoretical plates shall not be less than 3000 according to the chrysophane peak.

**2. Preparation of reference solution**

Take an appropriate amount of emodin reference substance dried under reduced pressure for 24 hours with phosphorus pentoxide as a desiccant. Add methanol to prepare a solution containing 20 µg per 1ml.

**3. Preparation of test solution**

Take about 0.1g of this product and add 25ml of chloroform and 20ml of 2.5mol/l sulfuric acid solution. Heat it in an 80 ℃ water bath for 2 hours, cool it to room temperature, weigh it again, make up the lost weight with chloroform, and shake it well. Separately take the chloroform solution, accurately measure 5ml, evaporate to dryness, add methanol to dissolve the residue, transfer it to a 20ml volumetric flask, add methanol to dilute to the scale, shake well, filter, and take the continuous filtrate.

**4. Determination method**

Accurately absorb the control solution and the test solution 10-20 μl respectively. Inject them into the liquid chromatograph for determination.

Calculated as dry product: the content of emodin (c15h10o5) per 1g shall not be less than 4.0mg.


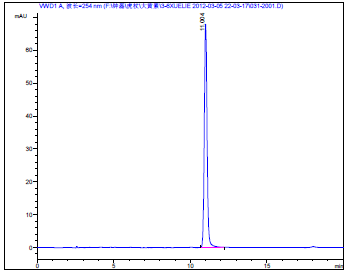


Figure 1. The HPLC of emodin


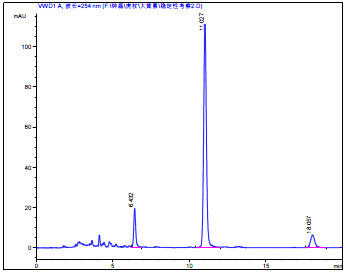


Figure 2. The HPLC fingerprint of polygoni cuspidati rhizoma et radix

**Cuscutae semen**

**1. Chromatographic conditions and system suitability test**

Octadecyl silane bonded silica gel was used as filler. Acetonitrile-0.1% phosphoric acid solution (17:83) is used as the mobile phase. The detection wavelength is 360nm. The number of theoretical plates shall not be less than 5000 according to hypericin peak.

**2. Preparation of reference solution**

Take an appropriate amount of hypericin reference substance and add methanol to make a solution containing 48 µg per 1ml.

**3. Preparation of test solution**

Take about 0.5g of this product under the item of difference in loading volume, grind it into fine pieces, weigh it precisely, place it in a conical flask with a stopper, add 50ml of 80% methanol precisely, weigh it, conduct ultrasonic treatment (power 250W, frequency 50KHz) for 1 hour, cool it, weigh it again, make up the lost weight with 80% methanol, shake it well, filter it, and take the filtrate.

**4. Determination method**

Accurately absorb the control solution 5-10 μl and the test solution 10μl. Inject them into the liquid chromatograph for determination.

Calculated as dry product: the content of hypericin (c21h20o12) per 1g shall not be less than 0.45mg.


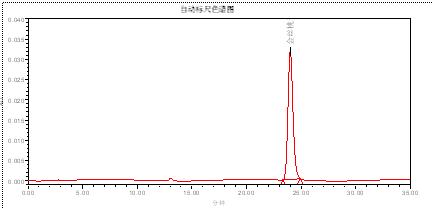


Figure 1. The HPLC of hypericin


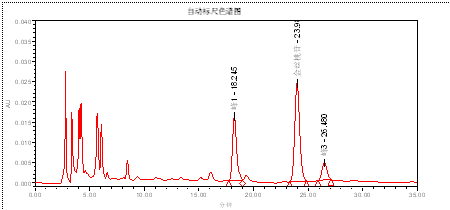


Figure 2. The HPLC fingerprint of Cuscutae semen
